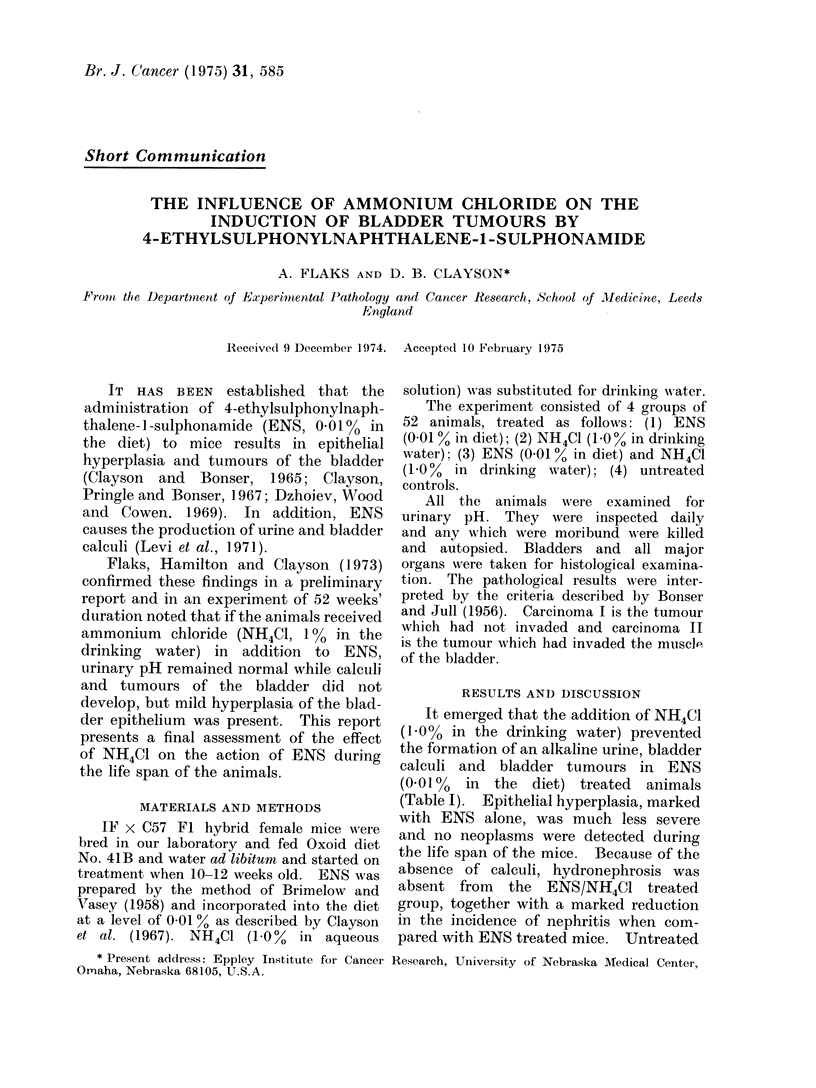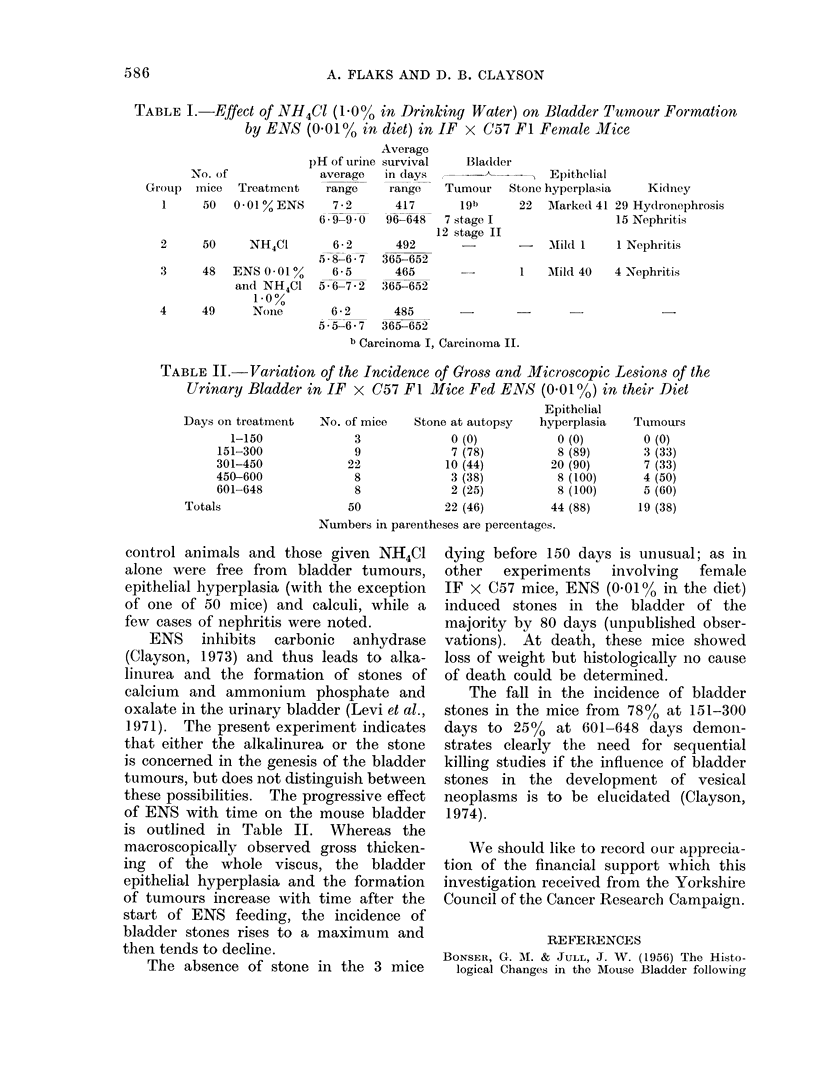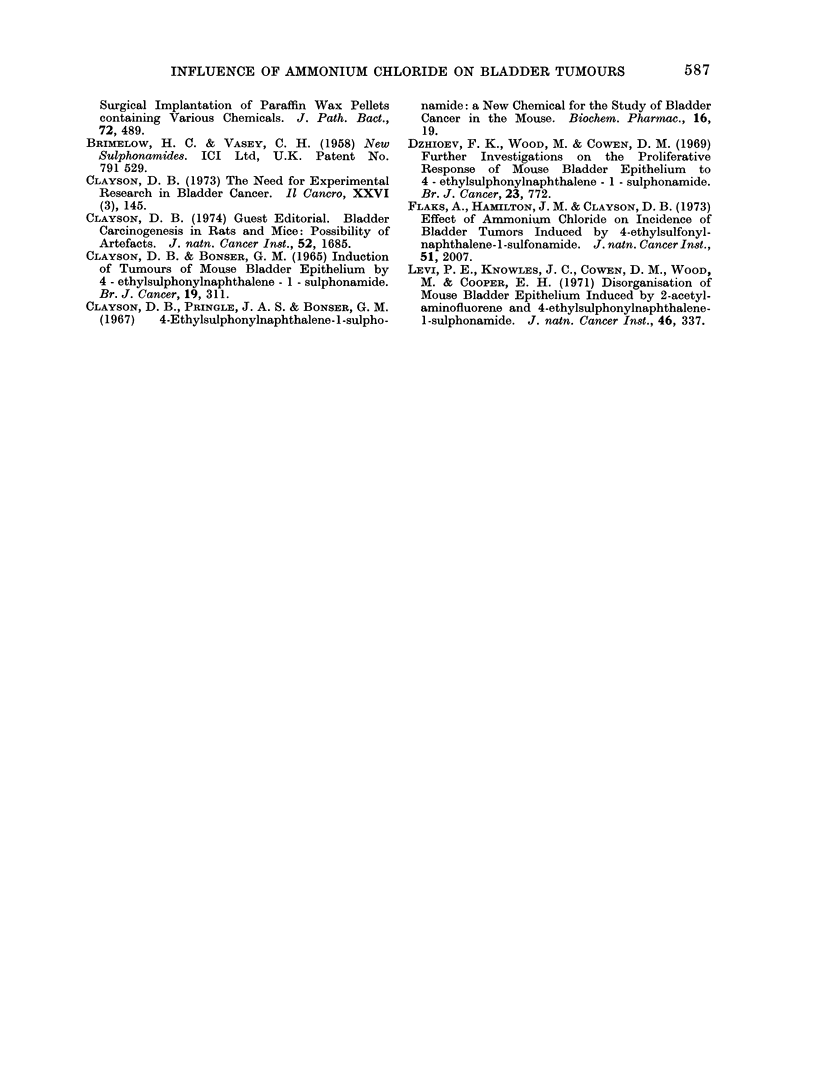# The influence of ammonium chloride on the induction of bladder tumours by 4-ethylsulphonylnaphthalene-1-sulphonamide.

**DOI:** 10.1038/bjc.1975.100

**Published:** 1975-05

**Authors:** A. Flaks, D. B. Clayson


*Br. J. Cancer* (1975) **31**, 585

***Short Communication***

### THE INFLUENCE OF AMMONIUM CHLORIDE ON THE INDUCTION OF BLADDER TUMOURS BY 4-ETHYLSULPHONYLNAPHTHALENE-1-SULPHONAMIDE

A. FLAKS AND D. B. CLAYSON*

*From the Department of Experimental Pathology and Cancer Research, School of Medicine, Leeds England*

Received 9 December 1974. Accepted 10 February 1975

IT HAS BEEN established that the administration of 4-ethylsulphonylnaphthalene-1-sulphonamide (ENS, 0·01% in the diet) to mice results in epithelial hyperplasia and tumours of the bladder (Clayson and Bonser, 1965; Clayson, Pringle and Bonser, 1967; Dzhoiev, Wood and Cowen, 1969). In addition, ENS causes the production of urine and bladder calculi (Levi *et al.*, 1971).

Flaks, Hamilton and Clayson (1973) confirmed these findings in a preliminary report and in an experiment of 52 weeks' duration noted that if the animals received ammonium chloride ($NH_4Cl$, 1% in the drinking water) in addition to ENS, urinary pH remained normal while calculi and tumours of the bladder did not develop, but mild hyperplasia of the bladder epithelium was present. This report presents a final assessment of the effect of $NH_4Cl$ on the action of ENS during the life span of the animals.

### MATERIALS AND METHODS

IF × C57 F1 hybrid female mice were bred in our laboratory and fed Oxoid diet No. 41B and water *ad libitum* and started on treatment when 10–12 weeks old. ENS was prepared by the method of Brimelow and Vasey (1958) and incorporated into the diet at a level of 0·01% as described by Clayson *et al.* (1967). $NH_4Cl$ (1·0% in aqueous solution) was substituted for drinking water.

The experiment consisted of 4 groups of 52 animals, treated as follows: (1) ENS (0·01% in diet); (2) $NH_4Cl$ (1·0% in drinking water); (3) ENS (0·01% in diet) and $NH_4Cl$ (1·0% in drinking water); (4) untreated controls.

All the animals were examined for urinary pH. They were inspected daily and any which were moribund were killed and autopsied. Bladders and all major organs were taken for histological examination. The pathological results were interpreted by the criteria described by Bonser and Jull (1956). Carcinoma I is the tumour which had not invaded and carcinoma II is the tumour which had invaded the muscle of the bladder.

### RESULTS AND DISCUSSION

It emerged that the addition of $NH_4Cl$ (1·0% in the drinking water) prevented the formation of an alkaline urine, bladder calculi and bladder tumours in ENS (0·01% in the diet) treated animals (Table I). Epithelial hyperplasia, marked with ENS alone, was much less severe and no neoplasms were detected during the life span of the mice. Because of the absence of calculi, hydronephrosis was absent from the ENS/$NH_4Cl$ treated group, together with a marked reduction in the incidence of nephritis when compared with ENS treated mice. Untreated

* Present address: Eppley Institute for Cancer Research, University of Nebraska Medical Center, Omaha, Nebraska 68105, U.S.A.

TABLE I.—*Effect of $NH_4Cl$ (1·0% in Drinking Water) on Bladder Tumour Formation by ENS (0·01% in diet) in IF × C57 F1 Female Mice*

| Group | No. of mice | Treatment | pH of urine average / range | Average survival in days / range | Bladder: Tumour | Bladder: Stone | Epithelial hyperplasia | Kidney |
|---|---|---|---|---|---|---|---|---|
| 1 | 50 | 0·01% ENS | 7·2 | 417 | 19[b] | 22 | Marked 41 | 29 Hydronephrosis |
| | | | 6·9–9·0 | 96–648 | 7 stage I | | | 15 Nephritis |
| | | | | | 12 stage II | | | |
| 2 | 50 | $NH_4Cl$ | 6·2 | 492 | — | — | Mild 1 | 1 Nephritis |
| | | | 5·8–6·7 | 365–652 | | | | |
| 3 | 48 | ENS 0·01% | 6·5 | 465 | — | 1 | Mild 40 | 4 Nephritis |
| | | and $NH_4Cl$ | 5·6–7·2 | 365–652 | | | | |
| | | 1·0% | | | | | | |
| 4 | 49 | None | 6·2 | 485 | — | — | — | — |
| | | | 5·5–6·7 | 365–652 | | | | |

[b] Carcinoma I, Carcinoma II.

TABLE II.—*Variation of the Incidence of Gross and Microscopic Lesions of the Urinary Bladder in IF × C57 F1 Mice Fed ENS (0·01%) in their Diet*

| Days on treatment | No. of mice | Stone at autopsy | Epithelial hyperplasia | Tumours |
|---|---|---|---|---|
| 1–150 | 3 | 0 (0) | 0 (0) | 0 (0) |
| 151–300 | 9 | 7 (78) | 8 (89) | 3 (33) |
| 301–450 | 22 | 10 (44) | 20 (90) | 7 (33) |
| 450–600 | 8 | 3 (38) | 8 (100) | 4 (50) |
| 601–648 | 8 | 2 (25) | 8 (100) | 5 (60) |
| Totals | 50 | 22 (46) | 44 (88) | 19 (38) |

Numbers in parentheses are percentages.

control animals and those given $NH_4Cl$ alone were free from bladder tumours, epithelial hyperplasia (with the exception of one of 50 mice) and calculi, while a few cases of nephritis were noted.

ENS inhibits carbonic anhydrase (Clayson, 1973) and thus leads to alkalinurea and the formation of stones of calcium and ammonium phosphate and oxalate in the urinary bladder (Levi *et al.*, 1971). The present experiment indicates that either the alkalinurea or the stone is concerned in the genesis of the bladder tumours, but does not distinguish between these possibilities. The progressive effect of ENS with time on the mouse bladder is outlined in Table II. Whereas the macroscopically observed gross thickening of the whole viscus, the bladder epithelial hyperplasia and the formation of tumours increase with time after the start of ENS feeding, the incidence of bladder stones rises to a maximum and then tends to decline.

The absence of stone in the 3 mice dying before 150 days is unusual; as in other experiments involving female IF × C57 mice, ENS (0·01% in the diet) induced stones in the bladder of the majority by 80 days (unpublished observations). At death, these mice showed loss of weight but histologically no cause of death could be determined.

The fall in the incidence of bladder stones in the mice from 78% at 151–300 days to 25% at 601–648 days demonstrates clearly the need for sequential killing studies if the influence of bladder stones in the development of vesical neoplasms is to be elucidated (Clayson, 1974).

We should like to record our appreciation of the financial support which this investigation received from the Yorkshire Council of the Cancer Research Campaign.

REFERENCES

BONSER, G. M. & JULL, J. W. (1956) The Histological Changes in the Mouse Bladder following

Surgical Implantation of Paraffin Wax Pellets containing Various Chemicals. *J. Path. Bact.*, **72**, 489.

BRIMELOW, H. C. & VASEY, C. H. (1958) *New Sulphonamides*. ICI Ltd, U.K. Patent No. 791 529.

CLAYSON, D. B. (1973) The Need for Experimental Research in Bladder Cancer. *Il Cancro*, **XXVI** (3), 145.

CLAYSON, D. B. (1974) Guest Editorial. Bladder Carcinogenesis in Rats and Mice: Possibility of Artefacts. *J. natn. Cancer Inst.*, **52**, 1685.

CLAYSON, D. B. & BONSER, G. M. (1965) Induction of Tumours of Mouse Bladder Epithelium by 4 - ethylsulphonylnaphthalene - 1 - sulphonamide. *Br. J. Cancer*, **19**, 311.

CLAYSON, D. B., PRINGLE, J. A. S. & BONSER, G. M. (1967) 4-Ethylsulphonylnaphthalene-1-sulphonamide: a New Chemical for the Study of Bladder Cancer in the Mouse. *Biochem. Pharmac.*, **16**, 19.

DZHIOEV, F. K., WOOD, M. & COWEN, D. M. (1969) Further Investigations on the Proliferative Response of Mouse Bladder Epithelium to 4 - ethylsulphonylnaphthalene - 1 - sulphonamide. *Br. J. Cancer*, **23**, 772.

FLAKS, A., HAMILTON, J. M. & CLAYSON, D. B. (1973) Effect of Ammonium Chloride on Incidence of Bladder Tumors Induced by 4-ethylsulfonylnaphthalene-1-sulfonamide. *J. natn. Cancer Inst.*, **51**, 2007.

LEVI, P. E., KNOWLES, J. C., COWEN, D. M., WOOD, M. & COOPER, E. H. (1971) Disorganisation of Mouse Bladder Epithelium Induced by 2-acetylaminofluorene and 4-ethylsulphonylnaphthalene-1-sulphonamide. *J. natn. Cancer Inst.*, **46**, 337.